# Inverted Covariate Effects for First versus Mutated Second Wave Covid-19: High Temperature Spread Biased for Young

**DOI:** 10.3390/biology9080226

**Published:** 2020-08-14

**Authors:** Hervé Seligmann, Siham Iggui, Mustapha Rachdi, Nicolas Vuillerme, Jacques Demongeot

**Affiliations:** 1 Laboratory AGEIS EA 7407, Team Tools for e-Gnosis Medical & Labcom CNRS/UGA/OrangeLabs Telecom4Health, Faculty of Medicine, University Grenoble Alpes (UGA), 38700 La Tronche, France; sihamiggui95@gmail.com (S.I.); Mustapha.Rachdi@univ-grenoble-alpes.fr (M.R.); Nicolas.Vuillerme@univ-grenoble-alpes.fr (N.V.); Jacques.Demongeot@univ-grenoble-alpes.fr (J.D.); 2 The National Natural History Collections, The Hebrew University of Jerusalem, Jerusalem 91404, Israel

**Keywords:** COVID-19, exponential slope, regression, random drift, adaptation for low pathogenicity

## Abstract

(1) Background: Here, we characterize COVID-19’s waves, following a study presenting negative associations between first wave COVID-19 spread parameters and temperature. (2) Methods: Visual examinations of daily increases in confirmed COVID-19 cases in 124 countries, determined first and second waves in 28 countries. (3) Results: The first wave spread rate increases with country mean elevation, median population age, time since wave onset, and decreases with temperature. Spread rates decrease above 1000 m, indicating high ultraviolet lights (UVs) decrease the spread rate. The second wave associations are the opposite, i.e., spread increases with temperature and young age, and decreases with time since wave onset. The earliest second waves started 5–7 April at mutagenic high elevations (Armenia, Algeria). The second waves also occurred at the warm-to-cold season transition (Argentina, Chile). Second vs. first wave spread decreases in most (77%) countries. In countries with late first wave onset, spread rates better fit second than first wave-temperature patterns. In countries with ageing populations (for example, Japan, Sweden, and Ukraine), second waves only adapted to spread at higher temperatures, not to infect the young. (4) Conclusions: First wave viruses evolved towards lower spread. Second wave mutant COVID-19 strain(s) adapted to higher temperature, infecting younger ages and replacing (also in cold conditions) first wave COVID-19 strains. Counterintuitively, low spread strains replace high spread strains, rendering prognostics and extrapolations uncertain.

## 1. Introduction

Spread parameters of the Covid-19 pandemic decrease with temperature [1]. This could be a direct effect of temperature causing faster aerosol evaporation, limiting travel time and distance of airborne droplets with viral particles. Alternatively, high temperature due to insulation is a proxy for ultra-violet light (UV) exposure. UVs are highly mutagenic and can decrease viral “viability”. Prediction or early detection of second waves could be valuable for policy decisions [2] and seems more accurate than usually believed [3]. The same is true for determining climatic conditions favorable to viral spread [4,5]. Surprisingly, comparisons among Italian regions show that in May, temperature increased viral spread, a pattern opposite to that observed in March [6]. Hence, this observation on Italian regions predicts that when comparing different countries, second wave spread parameters could increase with temperature.

## 2. Methods

We followed the same methodology as in [1] by using coefficients (slopes) from regression analyses, adjusting an exponential model y = a∗exp(b∗x) where y is the daily number of new confirmed COVID-19 cases, x is the number of days since wave onset, a is a constant, and b is the slope. This corresponds to the log-transformed version ln y = ln a + b∗x. Daily numbers of new cases and total numbers of tests per countries are from [7], data on mean elevation from [8], mean temperature from [9], and counts of mutations from [10,11]. For each country, population density is from [12] and median population age from [13].

Visual graph examinations, also called eyeballing, can produce spurious results because of arbitrariness in defining the start of the new trend [14], in this case a new increase in infection cases. Applying statistical models accounting for non-stationary patterns rather than eyeballing does not alleviate the problem of over-detection of changepoints in trends [15]. Solving this problem requires complex methods, mainly by applying simulations to the data [16,17].

We used a simplified statistical approach to test for visual detection of the start of the second wave. We set a moving time window size of 20 days. We calculated, for each period of 20 days, the Pearson correlation coefficient r between time and daily numbers of new cases and examined *r* as a function of the first day included in the moving window. Typically, high r values corresponding to a fast increase of the first wave are followed by a decrease in *r*. We searched for a second high (local maximum) *r*, which presumably indicated the onset date of the second wave according to this statistical method. Onset dates as determined visually and by this statistical method were compared.

Note that window size is the only arbitrary parameter of this approach. Results could vary according to window size, and the optimal window size could vary according to different datasets. Solving these specific problems, as well as the overall problem of breaking point detection, is beyond our scope. This would not contribute to the issue at stake and would detract attention from the urgent epidemiological and environmental aspects of the ongoing pandemic.

## 3. Results

### 3.1. Relationship between Covid-19 Cases and Mean Elevation

**Figure 1:**
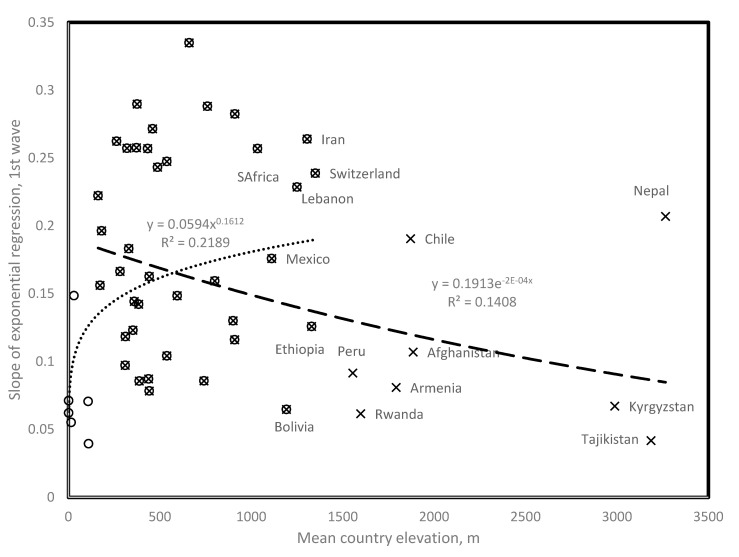
Slope of daily new confirmed Covid-19 cases as a function of mean country elevation. (Circles) Countries contributing to the positive trend with elevation, up to 1400 m, *r* = 0.468, two tailed *p* = 0.0018; (X) Countries contributing to the negative trend with elevation, down to 110 m, *r* = −0.375, two tailed *p* = 0.01. Note that for countries above 1000 m, landlocked or isolated countries tend to fit the negative trend (for example, Bolivia, Ethiopia, Armenia, and Afghanistan) as opposed to countries with large coastal populations (for example, Chile and South Africa) and landlocked Nepal and Switzerland probably contaminated by tourists from low elevation countries. Peru has a low slope and a large coastal population. Data are from Table 1.

Figure 1 plots slopes of exponential regressions on time of daily new cases (calculated as a function of days since first 100 confirmed cases) as a function of mean country elevation. Exponents (which estimate contagion rates) increase with temperature up to 900–1000 m, then, drop above 1000 m, especially for landlocked high elevation countries. This analysis potentially disentangles co-linearities between temperature and UV.

The trend below 1000 m confirms previously described effects of temperature on spread parameters, as temperature decreases with elevation. The drop in the exponents above 1000 m elevation indicates direct UV effects, probably by increasing deleterious mutations. These observations are for exponents estimated for the first Covid-19 wave, for each country (Table 1).

We use this pattern as preliminary evidence to justify the working hypothesis that changes in epidemiological patterns between first and second waves could be due to mutations.

### 3.2. Covid-19 Viruses Evolve Over Time

**Figure 2:**
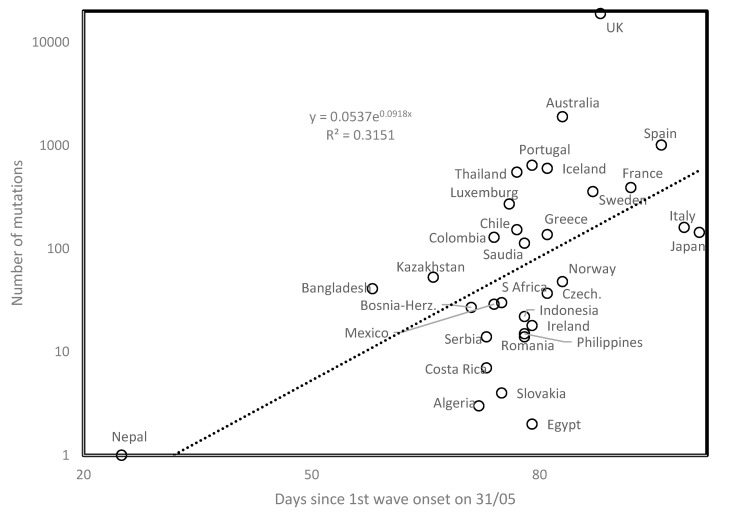
Mutation numbers as a function of days since onset of first wave (determined on 31 May).

The number of mutations in a country increases with time since first wave onset (*r* = 0.561, two-tailed *p* = 0.00084, Figure 2). Time since onset is indeed proportional to replicational cycles, and viral population evolution. No meaningful correlation was observed between mutation numbers and country mean temperature or elevation. The results remain qualitatively unchanged after excluding from analysis extreme datapoints (Nepal, London, UK).

Identical mutations sometimes occur in different populations of the Covid-19 virus [18], called parallel evolution. Close positions of African, Asian, European and South American countries with high elevation in Figure 1 potentially suggest parallel evolutions at high altitudes affecting spread parameters of these distant viral lineages. Strengthening this point, Georgia, which was not initially included in our sample, has a low first wave slope = 0.0346 with a mean altitude of 1432 m.

### 3.3. Determination of First and Second Waves

**Figure 3:**
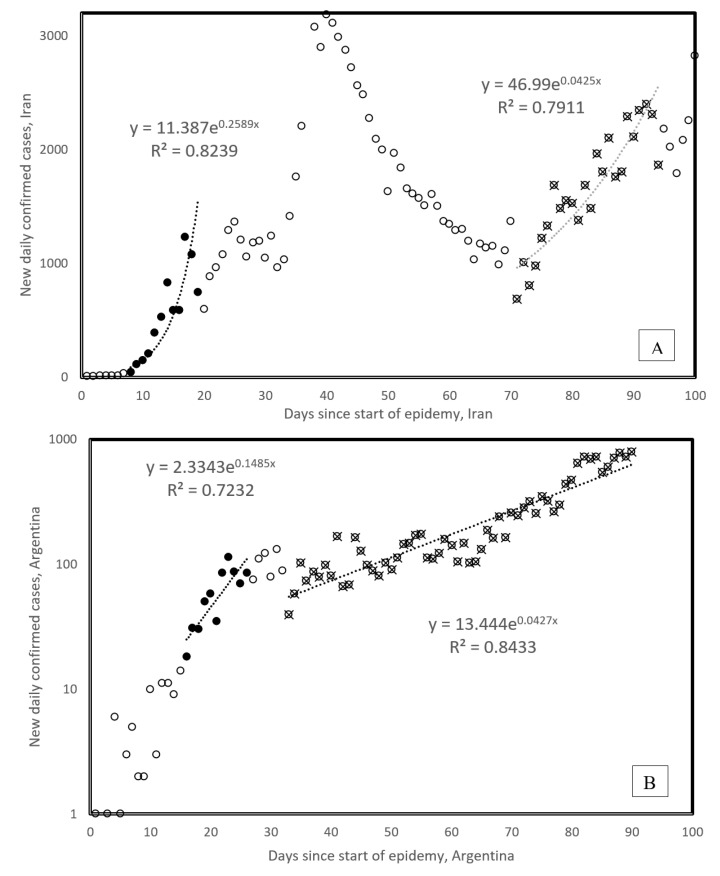
First and second waves of Covid-19 epidemy in Iran (**A**) and Argentina (**B**). First wave onsets are defined from the day the cumulative total number of confirmed cases passes 100 cases. Onset of second waves is determined visually. All countries, but Chile, follow the general pattern, as in the example for Iran, where the new wave follows a decrease; Chile follows the pattern of Argentina. Note the log scale for the Figure 3B *y* axis. This presentation mode was chosen in order to visually enhance slope change. Data are from Table 1.

Here, we study exponents estimated for second Covid-19 waves, derived from visually examining daily new cases in 123 countries. We explored temporal-, geographic-, demographic- and temperature-associated patterns of second wave spread parameters. We examined graphs plotting daily numbers of new confirmed cases (as daily updated at https://www.worldometers.info/ coronavirus/ [2]) for 123 countries. Second waves were visually determined, with examples in Figure 3 (Iran and Argentina). Second waves occurred in 26 countries, along patterns shown for Iran (broken first wave, second wave started from a low rate). The pattern shown for Argentina (new slope after inflection in first wave still in its growing phase (note the logarithmic scale of the *y* axis of Figure 3B)) occurs only in one other country, i.e., neighboring Chile. For Argentina and Chile, the new second wave slope occurred during the hot-to-cold season transition, in early April, corresponding to an early October northern hemisphere seasonal shift.

The lower second vs. first wave slopes in Figure 3 are not due to temperature increase, as could be expected from negative correlations between first wave slopes and temperature [1]. This is because for Argentina and Chile (Table 1), lower slopes correspond to hot-to-cold season transition, but not cold-to-hot seasons. Table 1 compares the first and second wave slopes.

**Table 1:** Exponential slopes of first and second Covid-19 waves in countries with two detected waves. Columns 1, Country; Column 2, T, mean annual temperatures; Column 3, E, mean elevation; Column 4, D, density; Column 5, A, median age in that country. Start S1 for first wave is the date when cumulated total confirmed cases reached 100, start S2 for second wave is visually estimated as in Figure 3. Numbers following second wave onset date indicate differences with onset dates determined by other methods, see Section 3.11 and Section 3.12. Slopes are the exponent b from the exponential regression y = a∗exp(b∗x), where y is the number of new daily cases and x the number of days since 100 cumulated cases for the first wave, or second wave start. First wave data were completed by data from [1] and countries with mean elevation >900 m (indicated with *). In Kenya and Sri Lanka, erratic data prevent estimating first wave slopes.

Country	T	E	D	A	1st wStart	S1	2nd wStart	S2
Africa								
Algeria	22.5	800	18	28.1	20/3	0.1594	07/4 −4	0.0316
Kenya	24.75	752	82	19.7			12/5 1 5	0.0740
Ethiopia *	22.2	1330	101	17.9	21/4	0.1259		
Morocco *	17.1	909	80	29.3	21/3	0.1161		
Rwanda *	17.85	1598	470	19.0	04/4	0.0615		
South Africa *	17.75	1034	48	27.1	17/3	0.257		
Asia								
Afghanistan *	12.6	1885	49	18.9	26/3	0.107		
Bahrain [1]	27.15	1	1983	32.3	09/3	0.1884		
Iran	17.25	1305	51	30.3	26/2	0.2641	01/5 −1 24	0.0438
Iraq	14.03	312	90	20.0	14/3	0.1184	15/4 1	0.041
Japan [1]	11.15	438	333	47.3	20/2	0.0872		
Kazakhstan	6.4	387	7	30.6	26/3	0.0856	08/5 −4 22	0.0933
Kyrgyzstan	1.55	2988	32	26.5	30/3	0.0671	25/4 3	0.0271
Lebanon	16.4	1250	672	30.5	14/3	0.2286	19/4 −2	0.0757
Malaysia [1]	25.4	538	99	28.5	08/3	0.1042	12/5 5 0	0.0794
Nepal *	8.1	3265	201	24.1	06/5	0.207		
Oman	25.6	310	15	25.6	25/3	0.0972	02/5 −3 7	0.0936
Pakistan *	20.20	900	274	23.8	15/3	0.1301		
Philippines	25.85	442	362	23.5	14/3	0.1627	22/5 7 9	0.1772
Singapore [1]	26.45	15	7894	34.6	28/2	0.0551	02/5 28 3	0.0641
South Korea	11.5	282	517	41.8	20/2	0.1664	06/5 −10 6	0.0585
Sri Lanka	26.95	228	332	32.8			08/5 0	0.1347
Tajikistan *	2.00	3186	64	24.5	02/5	0.0418		
Uzbekistan	12.05	353 ^$^	73	28.6	28/3	0.1231	26/4 1	0.0238
Australia [1]	21.65	330	3	38.7	09/3	0.1832		
Europe								
Armenia	7.15	1792	99	35.1	18/3	0.0809	05/4 −1 4	0.057
Austria [1]	6.35	910	106	44.0	08/3	0.2825		
Azerbaijan	11.95	384	116	32.3	25/3	0.1422	25/4 0	0.0676
Belgium [1]	9.55	181	378	41.4	06/3	0.1963		
Czech Rep. [1]	7.55	433	135	42.1	11/3	0.257	13/5 11 15	0.0474
Denmark				42.2				
France [1]	10.7	375	123	41.4	29/2	0.2898		
Germany [1]	8.5	263	233	47.1	29/2	0.2624		
Italy [1]	12.45	538	200	45.5	22/2	0.2475		
Lithuania	6.2	110	73	43.7	21/3	0.0394	05/5 −6 5	0.0554
Malta	19.2	1	1567	41.8	23/3	0.0712	19/4 −13 −10	0.0536
N Macedonia	9.8	741	81	37.9	21/3	0.0858	03/5 1	0.0528
Netherlands [1]	9.25	30	421	42.6	05/3	0.2485		
Norway [1]	1.5	460	17	39.2	09/3	0.2716		
Poland	7.85	173	123	40.7	14/3	0.1562	05/4 −21 −36	0.0094
Portugal	15.15	372	112	42.2	13/3	0.0301	09/5 −1 13	0.0431
Spain [1]	13.3	660	93	42.7	25/2	0.335		
Sweden [1]	2.1	320	23	41.2	05/3	0.2572		
Switzerland [1]	5.5	1350	208	42.4	04/3	0.2388		
UK [1]	8.45	162	280	40.5	04/3	0.2223		
North America								
Canada [1]	−5.35	487	4	42.2	10/3	0.2432		
Cuba	25.2	108	102	41.5	27/3	0.0706	17/5 −2 4	0.0517
El Salvador	24.45	442	319	27.1	10/4	0.0783	21/4 2 2	0.0535
Guatemala	23.56	759	162	22.1	09/3	0.088	01/5 −2 −1	0.1109
Panama	25.4	360	56	29.2	18/3	0.1443	19/5 5 7	0.1195
Mexico *	21.00	1111	64	28.3	18/3	0.1759		
USA [1]	8.55	760	34	38.1	02/3	0.2882		
South America								
Argentina	14.8	595	16	31.7	18/3	0.1485	05/5 3 20	0.0427
Bolivia *	21.55	1192	10	24.3	30/3	0.0647		
Brazil				32.6				
Chile	8.45	1871	23	34.4	15/3	0.1906	30/4 2 10	0.0586
Peru *	19.6	1555	25	28.0	29/3	0.0915		

^$^ from https://www.atlasbig.com/en-us/countries-average-elevation.

### 3.4. Geographical Second Wave Clusters

Visual data examinations such as in Figure 3 for 123 countries, on 31 May, detect second waves in 28 countries from four continents (Africa (2), Americas (North, 4 and South, 2), Asia (12) and Europe (8)). For Kenya and Sri Lanka, first wave slopes could not be determined (Table 1). Earliest second waves are from Armenia and Poland (5 April), and Algeria (7 April). Second waves are distributed into the following four geographic clusters (from earliest to latest): one spreading from the high elevation Eurasian plateau to surrounding countries (5/4 Armenia, 15/4 Iraq, 19/4 Lebanon, 25/4 Azerbaijan, Kyrgyzstan, 26/4 Uzbekistan, 1/5 Iran, 2/5 Oman, 8/5 Kazakhstan), a Central American cluster (21/4 El Salvador, 1/5 Guatemala, 17/5 Cuba, 19/5 Panama), a South American cluster (30/4 Chile, 5/5 Argentina), and a South-East Asian cluster (2/5 Singapore, 8/5 Sri Lanka, 12/5 Malaysia, 22/5 Philippines). For the three latter, geographically disconnected clusters, the earliest second waves are within a period of 11 days.

### 3.5. Second Wave Slopes versus First Wave Slopes

Second wave slopes are lower than first wave slopes for 20 among 26 countries (exceptions include Guatemala, Kazakhstan, Lithuania, Philippines, Portugal, and Singapore), a statistically significant majority (two tailed sign test, *p* = 0.0047). The mean second wave slope decreases by 43% as compared with the first wave slope.

### 3.6. Second Wave Spread Rates and Temperature

**Figure 4:**
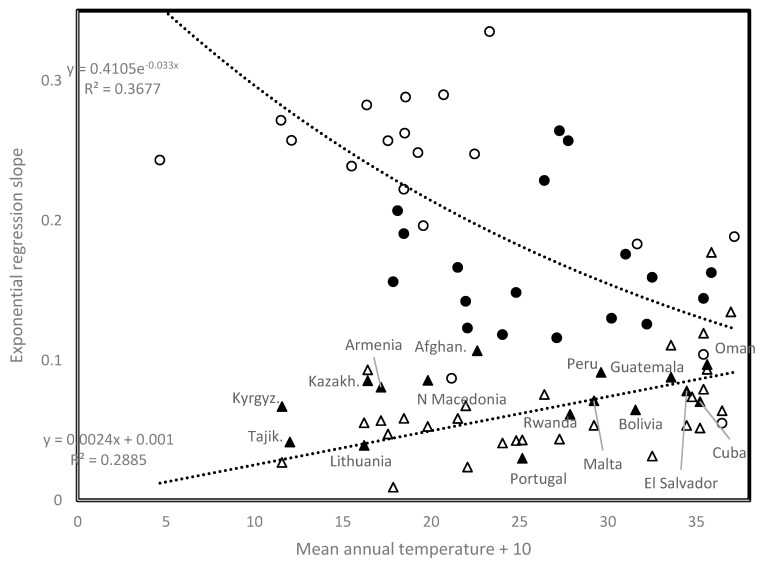
Slope of exponential regression of daily new cases vs. time, as a function of mean annual temperature, comparing trends for first wave slopes (open circles from [1], filled circles and filled triangles from the present study), and second wave slopes (open triangles). Data are from Table 1.

Figure 4 plots first and second wave slopes as a function of mean annual temperature. Analyses for the 16 countries from [1] show a negative association between first wave slope and mean annual temperature (open circles in Figure 4), producing *r* = −0.606, two tailed *p* = 0.0128. The overall pattern for the first wave (37 countries added, filled circles and filled triangles) remains qualitatively as in [1] (*r* = −0.329, *p* = 0.00817, one tailed test, considering all 53 countries).

Second wave slopes (open triangles, Figure 4) increase with temperature (*r* = 0.537, two tailed *p* = 0.00321). Unknown mechanisms enable second wave viral population spread at high temperatures. Earliest second wave occurrences at high elevations (Armenia, Algeria) may not be circumstantial. High UV regimes, increasing mutation rates, could occasionally favor selection of temperature-adapted viruses.

### 3.7. Time Since Start of First Wave for Low Slopes

For some countries, first wave slopes are closer to the regression line defined by second wave slopes than to the regression line defined by the first wave slopes. These countries are indicated in Figure 4 by filled triangles (second wave onset date before country): 9/3 Guatemala, 13/3 Portugal, 18/3 Armenia, 21/3 Lithuania, North Macedonia, 23/3 Malta, 25/3 Oman, 26/3 Afghanistan, Kazakhstan, 27/3 Cuba, 29/3 Peru, 30/3 Bolivia, Kyrgyzstan, 4/4 Rwanda, 10/4 El Salvador, 2/5 Tajikistan. On 31 May, the mean time since first wave onset in these countries was 65.19 days, significantly less than 76.32 days since first wave onset in remaining countries that fit best the negative trend (two tailed *t*-test, *p* = 0.0228). Hence, first wave viral population dynamics evolved with low spread in the latter.

### 3.8. Slopes and Times Since Start of First and Second Waves

**Figure 5:**
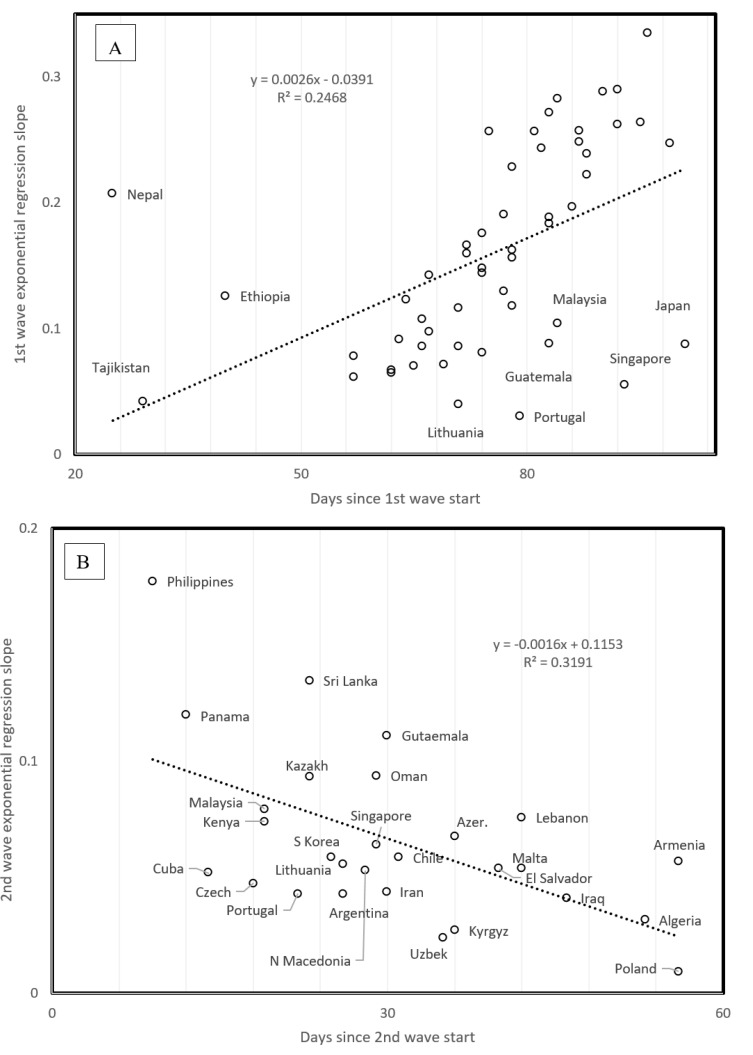
Slope of daily increase in confirmed COVID-19 cases as a function of time since wave started for (**A**) first wave and (**B**) second wave.

Time since first wave onset increases with spread slope (*r* = 0.4968, *p* = 0.00018, two tailed test, Figure 5A). Outliers with high slopes despite recent start associate with high elevation, outliers with low slope despite early first wave have developed marine commerce. Time since first wave start could be a proxy of temperature, as early first waves occurred in February vs. late ones that occurred in April. Seasonal temperature might decrease slopes at their start for countries with a late first wave. However, mean annual temperature across countries does not correlate with the time since first wave onset. Hence, the effect in Figure 5A seems independent of mean temperature.

This contrasts with patterns for the second wave, where time since second wave onset correlates negatively with second wave slope (*r* = −0.5649, *p* = 0.0026, two tailed test, Figure 5B). Hence, second wave viral populations could increase their spread over time, possibly implying adaptation.

### 3.9. Elevation and Population Density

Mean country elevation correlates negatively with time since first wave onset (*r* = −0.6095, *p* = 0.0000016, two tailed test). This suggests that the pandemic reached more elevated and possibly isolated countries later. Low elevation also associates with ports and probable spread via marine commerce.

Notable is that at this point, no pandemic property (Table 1) correlates with population density. One would have expected that slopes increase with population densities, but this is not the case (first wave, *r* = −0.1779 and *p* = 0.2068; second wave, *r* = 0.0128 and *p* = 0.94845, two tailed tests). It seems that most COVID-19 cases are in dense urban centers. These densities could vary among different cities, but mean country density does not reflect this. New York city and Singapore could have similar urban densities, but population densities for their respective countries vary due to size differences in surrounding low population areas. Hence, no correlation could be observed using our simple method.

### 3.10. Median Age and Spread Rates

**Figure 6:**
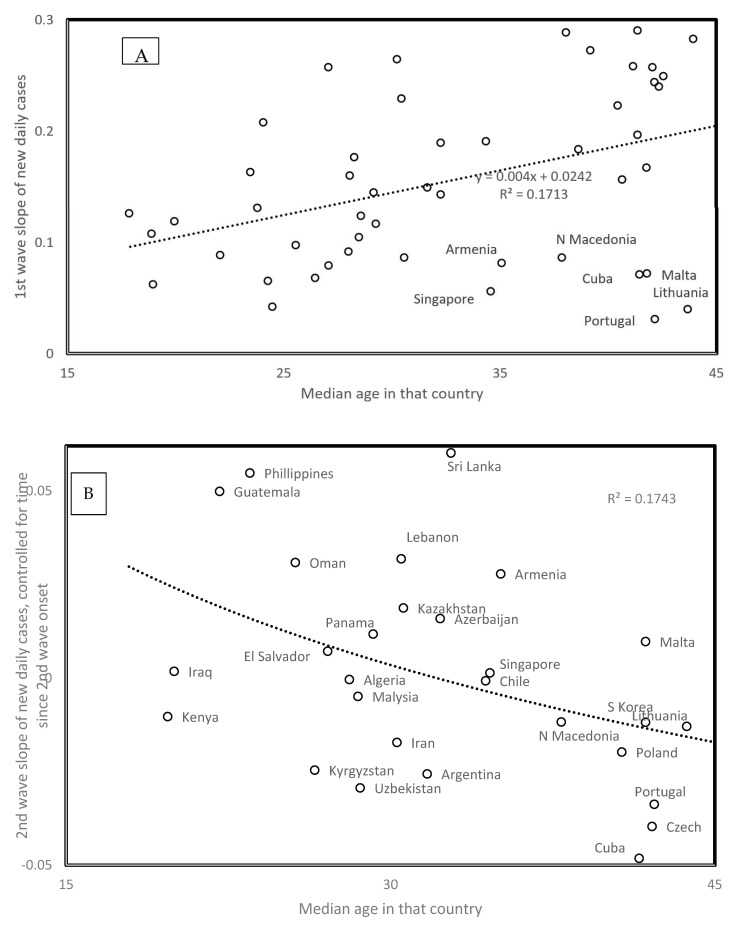
(**A**) First wave slope and (**B**) second wave slope, as a function of country median age. For second wave slopes, the figure plots the residual values after adjusting second wave slopes for time since second wave start (regression in Figure 4B), which corresponds to the main correlate of second wave slopes. Data are from Table 1.

First wave virus strains mainly hit the elderly. Hence, the positive correlation between slope and median population age in Figure 6A (*r* = 0.414, one tailed *p* = 0.0011) fits the expected higher contagiousness in ageing populations. Note outliers as indicated in Figure 6A. For the second wave, the opposite association occurs, i.e., slopes are the highest for countries with low median age (*r* = −0.418, two tailed *p* = 0.0023, Figure 6B). This new information is crucial for future management of the pandemic. Second wave viruses apparently adapted to infect the larger reservoir of potential younger hosts, in addition to adapting to spread at higher temperatures.

Data gathered until mid-June find second waves in additional countries. For countries with median ages above 36 years (Bulgaria, Japan, Moldova, Serbia, Sweden, and Ukraine), the trend for these second wave slopes fits that observed for the first wave slopes as a function of median age in Figure 6A. This indicates that in these countries with late second wave onset and high median age, viruses only adapted to seasonal temperature increase, but not to the relatively few young in these populations.

In some countries, the second wave could be an artefact due to sudden policy changes such as increasing daily test numbers, which increase numbers of new reported cases, but do not reflect any epidemiological change. Other second wave slopes estimated after 31 May fit the trend in Figure 6B. This is the case for Bahrain, the Democratic Republic of Congo, Ghana, Guatemala, Iran, Israel, and Jordan. These patterns could be explained by policy differences between countries. Our interpretation remains biological and suggests that viruses evolve in relation to host populations and climatic conditions because country-specific sampling artefacts are unlikely to produce overall patterns across countries such as those in Figure 5 and Figure 6.

### 3.11. Eyeballing versus Statistical Evaluation of Second Wave Onset Date

Second wave detection by visual examinations (eyeballing) has an arbitrary component. However, statistical methods mimicking the process underlying visual detections, which assume the onset of a new wave could occur any day, are biased for false positive detections [14,15]. In addition, the size of the moving window used in these methods is also arbitrary, which can lead to false negatives if the exponential increase period of the new wave is much shorter than the period of the window. Analyses accounting for different window sizes in different countries are unrealistic and beyond the scope of our analyses.

**Figure 7:**
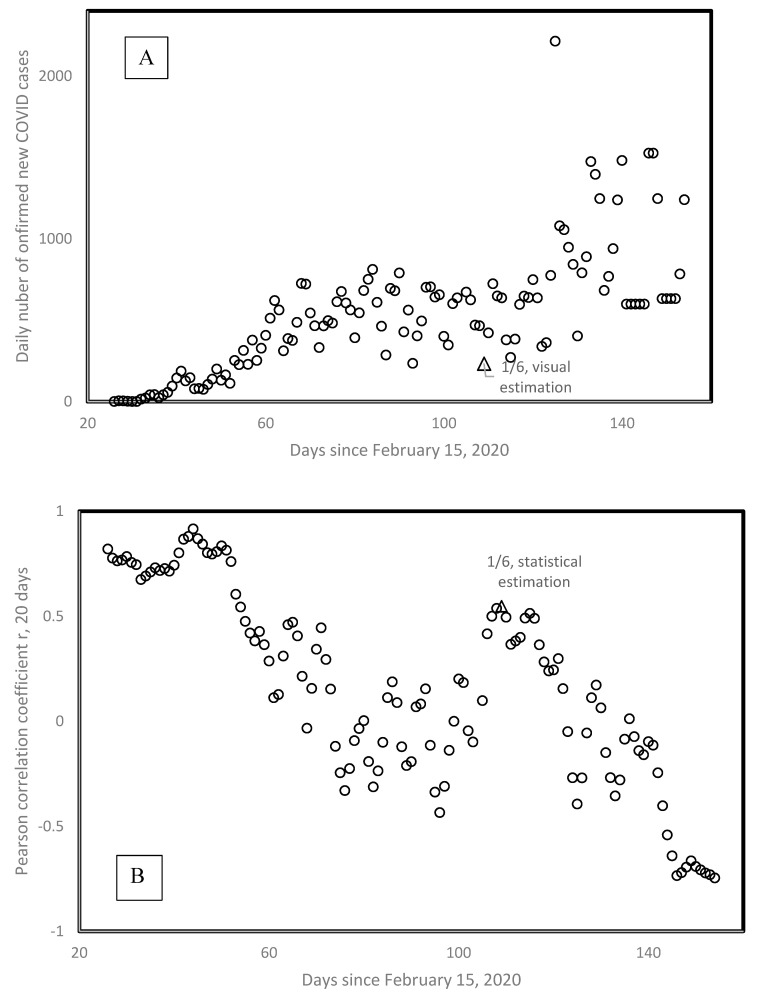
(**A**) Number of daily new confirmed cases in Sweden as a function of time since February 15; (**B**) Pearson correlation coefficient r for a running window of 20 consecutive days in (**A**), calculated for the whole period presented in (**A**), as a function of the days corresponding to the first date included in the running window. Visual examination of (**A**), and the local maximum of r in (**B**) define second wave onset at the same date, 1 June.

Figure 7 presents the visual and statistical estimation of the onset date of the second wave for Sweden. Visual estimation considers the minimum preceding a clear increase pattern in daily new confirmed cases, indicated by a triangle. This is the first of June (1/6), one day after we stopped our initial sampling of second waves. Figure 7B plots the Pearson correlation coefficient r calculated between the number of days since February 15 and the daily number of new confirmed cases for Sweden, the very data from Figure 7A. Calculations are done for a moving window of 20 consecutive days, and r is plotted as a function of the first day included in that moving window. Figure 7B shows that the highest rs are for the first wave, at the beginning of the epidemy in Sweden. Then r values decrease and increase again towards a second local maximum of *r* = 0.55, on 1 June.

Patterns from Figure 7A are typical and show how relatively little differences exist between visual and statistical estimations for the onset of the second wave. The first value following second wave onset dates in Table 1 indicate the numbers of days between the date set by eyeballing and that set by the statistical method. A value of −1 means that eyeballing determined the onset day one day earlier than the presumably more objective method using moving windows. Shifts between dates set by both methods are random. In half of the cases, eyeballing detects earlier, and the other half later second waves than the moving window method. Most shifts (77%) are small, between −5 and +5 days.

Hence, eyeballing is not biased as compared with an objective method for detecting second waves. However, eyeballing has the advantage that it does not arbitrarily set a priori the window size for detection of second waves, but rather adjusts its detection between two clear extreme dates during which a more or less monotonous increase in daily new cases occurred. In addition, eyeballing is rapid and simple. More formal statistical methods estimating P values of non-stationarity (meaning a new phenomenon in the time series) based on Monte-Carlo approaches require heavy computational efforts, justified in much more messy data, such as climatological data [14,15].

### 3.12. Total Numbers of Tests

An important caveat to our analyses is that for each country, they assume equal sampling effort across the whole period under study. However, numbers of tests vary across periods, hence an increase in the number of confirmed cases could result from an increase in tests, rather than from a second wave. The former is a sampling artefact, whereas the second is a natural phenomenon.

For that reason, we used total daily numbers of tests done for each country for which these data were available for the relevant period at [7] for countries for which Table 1 has second waves, and repeated visual examinations for daily percentages of positive tests among all tests done that day. The second value following second wave onset dates in Table 1 indicates differences in numbers of days between onset dates determined by eyeballing percentages vs. numbers of positive cases. Value −1 indicates that eyeballing numbers of positive cases detect an onset date that is earlier by one day than eyeballing percentages of positive cases. A bias exists in these differences, i.e., using numbers of positive cases detects later onsets dates in 16 among 19 countries where both approaches produced different dates. This is a statistically significant majority of countries according to a one-tailed sign test (*p* = 0.0022).

This suggests that using more information (number of positive cases and total number of tests) enables earlier detection of the phenomenon than when using only numbers of positive cases. In addition, because percentages detect earlier second waves, this means that increases detected according to numbers of positive cases did not produce false positives, but rather false negatives for the period that second waves remained undetected when using numbers rather than percentages. This indicates that increases in testing efforts do not occur independently of onsets of second waves. We suggest that medical experts probably sense very early on a change in the kinetics and increase testing efforts at these periods.

## 4. Discussion

Analyses confirm that the spread of first wave COVID-19 decreases with temperature. They indicate that UVs also decrease the spread of first wave COVID-19. Second wave COVID-19 is characterized by a lower spread and by infecting younger age classes. Second wave spread increases with temperature.

This inversion of trends between first and second waves, at one to two months interval, is highly peculiar. The possibility that a different virus was cryptic and minor during the first wave and became dominant as conditions changed during the second wave, cannot be excluded. However, trends with time and mutation numbers suggest that a specific virus evolved from one state to another. The earliest second waves, in high elevation countries, suggest UV-induced high mutation rates hastened adaptation. Adaptations could independently arise in different virus populations [18].

Alternative explanations relate to human behaviors and policies. Negative trends of spread with temperature in winter and positive ones in spring could reflect tendencies to stay inside in winter, and during warmer weather. Trends with population age could also be explained by seasonal differences in behaviors of different age groups. However, this would imply different complex explanations for each of the three independent pattern inversions described here, with temperature, population age, and time since wave onset. Non-random mutations could channel changes of viral RNA between two local structural optima, as described for COVID-19 in [19], one putatively adapted to cold and one to warm weather. This is more parsimonious than assuming different explanations for each of the three correlations reported here. This mutation hypothesis is also in line with observations that the earliest second waves usually occur at high altitudes where UVs could increase mutation rates.

This does not exclude the possibility of combined effects of mutations and behavioral changes in human populations (more alerted authorities and public adapting their behaviors), as well as fewer susceptible hosts (most infection-prone individuals are already been infected). However, the most parsimonious explanation is also likely to be the main factor in the case of a combined factor scenario.

Note that analyses determined clear patterns in relation to various cofactors of the pandemic, despite uncertainties in data. For example, reported over unreported cases ratio [20] apparently vary hugely between countries depending on their mode of counting and public health policy, rendering predictions for the future of the pandemic highly uncertain. A striking major point is unexplained and open for optimistic interpretations from a health-oriented point of view. Usually, strains with high spread replace those with low spread. However, low spread second wave viruses replace fast spread first wave viruses, in an increasing number of countries. This could suggest that third wave spread could further decrease. Another counterintuitive point is that for the sample of examined countries, viral spread does not increase with population density. Hence, accepted knowledge in relation to epidemics seems inadequate regarding the current pandemic. Hence, prognostics and interpretations of observed patterns, whether pessimistic or optimistic, cannot be trusted, as these are based on previous knowledge contradicting the current fast-to-slow spread evolution of COVID-19.

## 5. Conclusions

Current analyzes suggest that a third wave will occur with a possibly lower spread than for the first and second waves. A study is currently in progress to study its characteristics, in particular the correlations with the geo-climatic and demographic variables highlighted in [1] and in this article.
